# Remote Insects Trap Monitoring System Using Deep Learning Framework and IoT

**DOI:** 10.3390/s20185280

**Published:** 2020-09-15

**Authors:** Balakrishnan Ramalingam, Rajesh Elara Mohan, Sathian Pookkuttath, Braulio Félix Gómez, Charan Satya Chandra Sairam Borusu, Tey Wee Teng, Yokhesh Krishnasamy Tamilselvam

**Affiliations:** 1 Engineering Product Development Pillar, Singapore University of Technology and Design (SUTD), Singapore 487372, Singapore; rajeshelara@sutd.edu.sg (R.E.M.); sathian_pookkuttath@sutd.edu.sg (S.P.); brauliofenixgomez@hotmail.com (B.F.G.); sairambcsc007@gmail.com (C.S.C.S.B.); heyitsrafe@gmail.com (T.W.T.); 2 Department of Electrical Engineering, University of Western Ontario, London, ON N6A 3K7, Canada; ykrishn4@uwo.ca

**Keywords:** insects detection, CNN, deep learning, object detection, IoT, remote insect monitoring

## Abstract

Insect detection and control at an early stage are essential to the built environment (human-made physical spaces such as homes, hotels, camps, hospitals, parks, pavement, food industries, etc.) and agriculture fields. Currently, such insect control measures are manual, tedious, unsafe, and time-consuming labor dependent tasks. With the recent advancements in Artificial Intelligence (AI) and the Internet of things (IoT), several maintenance tasks can be automated, which significantly improves productivity and safety. This work proposes a real-time remote insect trap monitoring system and insect detection method using IoT and Deep Learning (DL) frameworks. The remote trap monitoring system framework is constructed using IoT and the Faster RCNN (Region-based Convolutional Neural Networks) Residual neural Networks 50 (ResNet50) unified object detection framework. The Faster RCNN ResNet 50 object detection framework was trained with built environment insects and farm field insect images and deployed in IoT. The proposed system was tested in real-time using four-layer IoT with built environment insects image captured through sticky trap sheets. Further, farm field insects were tested through a separate insect image database. The experimental results proved that the proposed system could automatically identify the built environment insects and farm field insects with an average of 94% accuracy.

## 1. Introduction

Environmental maintenance is an essential domain in all urban centers to control various pests and insects. Generally, lizards, crawling and flying insects such as cockroaches, ants, flies, etc. are common in any built environment (human-made physical spaces such as homes, hotels, camps, parks, pavement, hospitals, food industries etc.). These insects and lizards may create health hazards like allergy, asthma, food contamination illnesses, etc. Apart from health risks, economic loss due to insects is very high in the agriculture field and food industries. It threatens food supply at all stages, crop cultivation, processing, storage, and distribution. Early insect identification is crucial for effective and affordable control in urban environments, agriculture fields, and food process industries. It will help protect humans from health issues, prevent economic loss on the food industry, and reduce the excessive use of various harmful pesticides in agriculture.

Generally, pest management companies use manual inspection methods to monitor the insect population and control tasks accordingly. It is a time-consuming task and requires enormous human resources to handle the high-density urban area and large-sized agriculture farms effectively. Furthermore, workforce shortage is a critical issue in the insect control industry, mainly due to health issues, work in complex environments (manhole, sewer networks), and low wages [1]. Reports [2,3] indicate that other than identifying insects and handling various pesticides, this kind of job also needs sound knowledge in insect biology to manage pest control effectively.

A remote monitoring system is an emerging technique. In remote monitoring scheme, Internet of Things (IoT) is widely used method for various inspection applications, including health care [4], modern farming [5,6], human surveillance, environmental monitoring [7,8], object tracking in smart city [9,10] etc. Through IoT, the insect control team can monitor the insects trap anywhere on the globe.Potamitis et al. [11] proposed an IoT based smart trap monitoring system for controlling the crop insects. The IoT system was designed to collect real-time crops pest population information on the regional level and Global Positioning System (GPS) location. The collected data was sent to the server present at the Pest Management System (PMS), which will help to PMS for timely control of the pest population on the crop field. Rustia et al. [12] use the IoT network and wireless imaging system to develop the remote greenhouse pest monitoring system. The imaging system uses k-means color clustering and blob counting algorithm to automatically count the insect on the trap sheet. In another case study [13], IoT enabled smart farm field management scheme was proposed to continuously monitor crop growth, detect insects on the farm, and find suitable pesticide for control crop pests. The automated remote imaging system was proposed by Dusty et al. [14] for crop protection where Spensa Z-Trap, ADAMA trap view, and DPIRD moth trap module were used to monitor insect populations of farm field remotely. Eliopoulos et al. [15] developed an IoT-enabled smart trap for detecting crawling insects and arthropods in an urban environment. The trap comprises Infra-Red (IR) sensor, Complementary Metal–Oxide–Semiconductor (CMOS) camera sensor, and Wi-Fi module. Here, the IR sensor is used to trigger the camera whenever the insect enters the trap sheet and Wi-Fi to deliver the monitoring unit’s picture. In [16], Ilyas et al. proposed an IoT enabled electronic e-trap for fruit flies monitoring system. Here, the insect’s wing beats spectral content is used to count the number of insects on the trap. The bimodal optical sensor is used in electronic e-trap to measure the wing beats spectral content, and the General Packet Radio Service (GPRS) module was adopted for remote surveillance of insects. However, automatically identifying the insects or pests is another challenge in remote trap monitoring schemes.

Machine Learning (ML) and Deep Learning(DL) based object detection and decision-making are widely applied in various field [17,18,19], including insect control [20,21]. Irineo et al. [22] developed Integrated Pest Management (IPM) using a computer vision technique. The authors use LOSS v2 and Scales Invariant Feature Transform (SIFT) algorithms for detecting the various class of pest include from Diabrotica, Lacewings, Aphids, Glassy, Thrips, and Whitefly from the image. Image-based automated orchard insect identification and classification were proposed by Chenglu et al. [23], where the author using the global, local, and hierarchical features of insects for the train classifier framework. The evaluation results reveal that a hierarchical feature-based trained model obtained optimal classification results than local and global feature schemes. Chengjun et al. [24] proposed a field crop insect recognition method based on multiple task sparse representations and Multiple-Kernel Learning (MKL) algorithms. The method was tested with 24 insect classes and scored an average of 97% classification accuracy. YAO et al. designed the rice light-trap insect imaging system for monitor and control the rice pest population [25]. Here, a Support Vector Machine (SVM) classifier with a radial basis kernel function was used to identify the rice pest and train it with color, shape, and texture features. Jeric et al. [26] proposed a multi-class insect identification algorithm. Here, the authors use an unsupervised data collection technique to collect the insect image database and use the You Only Look Once v3 (YOLO v3) object detection algorithm to detect and automatically count the number of insects on a trap. Ding and Taylor [27] reported automatic moth detection from trap images. The authors use deep learning frame work for identifying and counting pests on the trap sheet images. The scheme uses a multi-layer Convolutional Neural Network (CNN) for feature extraction and a sliding window algorithm to detect insects from the extracted feature map. Liu et al. [28] developed a paddy field pest classification using Deep Convolutional Neural Network (DCNN) and Saliency Map. Here, AlexNet CNN architecture was customized for pest classification and localization. The network was trained with 5000 insect images and scored mean Average Precision (mAP) of 0.951.

Liu et al. [29] developed a DL based framework ’PestNet’ for large scale multi-class pest detection. The PestNet was trained with 80,000 pest images and obtained 75.46% mAP for multi-class pest detection. Nguyen and Phan [30] developed insect detection scheme on traps using DCNN. The author uses the Visual Geometry Group 16 (VGG16) CNN framework for feature extraction and Single Short Detector (SSD) object detection algorithm for detecting the insects on the trap. The CNN framework was trained with 3000 insect images and obtained 84% detection accuracy. Xia et al. [31] proposed an improved CNN framework to agricultural insect detection and classification where VGG19 and Region Proposal Network (RPN) modules were combined and trained with 4800 insect images. The experimental results indicate that the model took 0.083 s inference time and scored mAP of 0.8922. Gutierrez et al. [32] evaluated the efficiency of computer vision, machine learning, and deep learning algorithms for pest detection in tomato farms. The evaluation study indicates that the deep learning framework provided a comparatively better solution among the three options.

As mentioned above, the studies indicate that IoT is a more suitable technique for remote trap monitoring schemes. A DL framework is an optimal method to detect and classify insects through images. This work combines the IoT and DL framework to establish the remote insect trap monitoring and insect detection system. The system has been designed to detect the built environment lizard, crawling, and flying insects such as cockroaches, ants and flies, and selected farm field insects (Planthoppers, Colorado, Empoasca, Mole-cricket, Manduca, Rice Hispa, Stink-bug, and Whiteflies). This paper is organized as follows; after providing an introduction, motivation, and literature review in Section 1, Section 2 present the overview of the proposed system. Section 3 discusses the experimental setup, results, and discussion. Finally, Section 4 concludes this research work.

## 2. Proposed System

**Figure 1:**
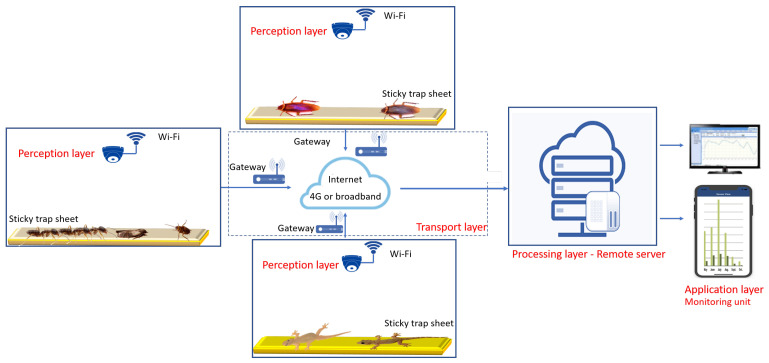
Overview of Internet of Things (IoT) based insect detection system.

Figure 1 shows the outline of IoT and DL based remote trap monitoring and insect detection framework. Four-layer IoT is used for constructing the remote trap monitoring system [33,34,35]. It comprises of perception layer, transport layer, processing layer, and application layer. The DL framework is trained and runs on the processing layer to carry out the remote insect identification task. The detail of IoT architecture and configuration of DL and other IoT component detail is described as follows.

### 2.1. Perception Layer

The perception layer is an end node or lowest layer in a four-layer IoT framework. It is constructed with smart wireless cameras and sticky type insect trap sheet. The camera was tiny and required very low power. It can fix on the roof or medium or large size food storage container for continuously monitored the insects. The Perception layer sends an insect trap images to the processing layer through the transport layer communication protocols to establish the remote insect monitoring and identification task.

### 2.2. Transport Layer

Transport layer act as a communication bridge between all the IoT layers. It accomplishes the following task: Sending the insect trap image to the processing layer and transferring the processed data to the application layer. The transport layer uses the WiFi communication and Transmission Control Protocol/Internet Protocol (TCP/IP) transport protocol for sharing the information.

### 2.3. Processing Layer

A processing layer is a central unit in our remote insect trap monitoring and detection system. It comprises high-speed computing devices for handling the image and video frame, executing the insect identification algorithm, and processing the application layer request. The detail of insect detection and classification framework is described as follows.

#### Insects Detection Module

A deep learning-based object detection algorithm is trained to identify the insects on the trap. Generally, most of the insects are small in size. So, insect detection algorithms need a small object detection capability. In terms of detecting insect presence in an image, the insects cover only a small number of pixels in an image, i.e., the area of interest in an image when detecting an insect is very small.

In any object detection algorithm, extracting the features from small objects is particularly challenging because there is a good chance that smaller objects might get overlapped or too pixelated to extract any useful information. Moreover, information extracted from smaller objects could be lost as it is passed on through multiple layers of feature extractor [36]. Furthermore, the information extracted about smaller objects is very limited as these objects appearance is very restricted in a training image. Small objects’ limited appearance leading to an incomprehensible feature extraction process could contribute to additional errors in detection results.

Due to various challenges discussed earlier in processing and detecting smaller objects in an image, insect detection needs an optimal feature extractor and detection algorithm, far more extensive, accurate, and apt for detecting smaller objects such as insects.

**Figure 2:**
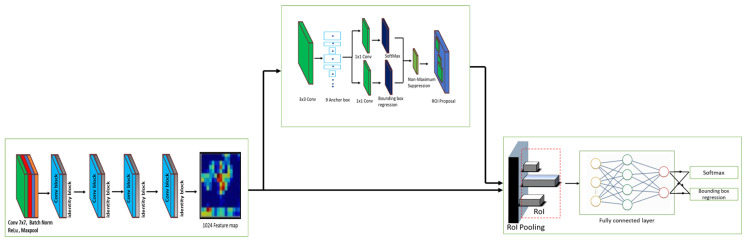
Faster Region-based Convolutional Neural Network (RCNN) RestNet architecture.

In this work Faster Region-based Convolutional Neural Networks (RCNN) RestNet 50 [37,38] is used for insect detection and classification task. Here, Faster RCNN is a two-stage object detector framework comprise of RPN and Fast RCNN module. It takes two shots to detect the object from the image. The first stage extracts the feature map and generating the region proposals, then the next step detecting the object from each proposal. Faster RCNN RestNet 50 is a unified deep learning based object detection approach. It performs both object detection and classification task. Figure 2 shows the block diagram of the Faster RCNN Residual neural networks 50 (ResNet50) framework. The unified DL framework consists of three modules, including ResNet 50, RPN, and Fast RCNN. Here, ResNet50 [39] is a pre-trained deep CNN algorithm that performs the feature extraction task and generates 1024 size feature maps. It’s an optimal algorithm for small object features extraction task. RPN is a fully connected small CNN network that produces the multiple regions of proposals and their objectness score from ResNet generated feature map. The final component is a Fast RCNN module that extracts the more features from the RPN proposed regions and output the bounding box and class labels. The detail of each module and its described as follows.

**Feature extractor:** Pretrained ResNet50 (trained on image-net) module is used in Faster RCNN object detection for feature extraction instead of VGG16. Unlike VGG 16 [40], VGG 19, ResNet is a much deeper network as the desired detection in this paper is much more complex than a regular image detection process. As the network becomes deeper, there is also a possibility of over-fitting, and therefore, a regularization process is done to eliminate any over-fitting on the training data. Moreover, the ResNet50 model uses average pooling layers, which reduces the data’s spatial size, further reducing the computational time and complexity of the algorithm. The model size is reduced down to 102 MB for ResNet50.

The feature extraction module consists of 5 stages. The first stage consists of 7 × 7 convolution, batch normalization, rectified linear unit (ReLu), and max-pooling operation. The next four stages consist of a combination of residual convolution block and identity block. Here, the residual conv block comprises of series of three convolution layers (1×1, 3×3, 1×1) with Batch Normalization (BN) and ReLu, and skip connection with 1×1 convolution operation. Similarly, identity blocks comprise of three convolution layer include 1×1, 3×3, 1×1, and skip connection. The fifth stage Conv and identity block output is a 1024 size feature map, which serves as input to the RPN module and Fast RCNN detection network.

Regional Proposal Network (RPN) In this unified DL framework, RPN is a crucial component that uses the anchor box technique to generate the bounding box on the output image. Anchor boxes are a set of predefined bounding boxes which has a certain height and width. Anchor boxes detect multiple objects, objects of different scales, and overlapping objects. The size of the anchor box is automatically chosen based on object size in the training dataset. For generating the anchor boxes and 512 size feature map, RPN took the final feature map (ResNet generated feature map) as input and applied a 3×3 sliding window spatially. For each sliding window position, nine anchor boxes are generated based on the sliding window’s center point. Those anchor boxes are three different ratios and three different scales. If the feature extraction layer’s final feature map has width W and height H, then the total number of anchors generated will be W*H*k. In the output side, an anchor box’s actual position in image level is determined by decode (by applying 16 as the stride in generating anchor boxes at image level) the feature map output back to the input image size. This process produces a set of tiled anchor boxes across the entire image. For each anchor, RPN predicts two things: The first is the probability that an anchor is an object (check whether the object is foreground or background and does not consider which class the object belongs to). Second is the bounding box regression for adjusting the anchors to fit the object better or similar to the ground truth. RPN used a 512 dimension feature map to carry out this operation and applied 1×1 convolutional operation to generate the low latitude vector. This low latitude vector is feed into the classifier layer and regression layer to predicts the above two things. Finally, Non-Maximum suppression (NMS) is applied to the generated proposal and filtering out the overlapping boxes.

Detection Network The second and final stage of Faster RCNN is a detection network where Fast RCNN components are adopted. The detection network comprises of Region of interest (ROI) pooling layer and a fully connected layer. The proposals generated by the RPN and the shared convolutional features are fed into the RoI pooling layer. RoI pooling layer extracts the fixed-size feature map for each RPN generated proposal. These fixed-size feature maps are forward to a fully connected layer with a softmax network and a linear regression function to classify the object and predict the bounding box for the identified objects. For accurate object localization, NMS (Non-Maximum suppression) is applied to remove redundant bounding boxes and retain the best one.

### 2.4. Application Layer

**Figure 3:**
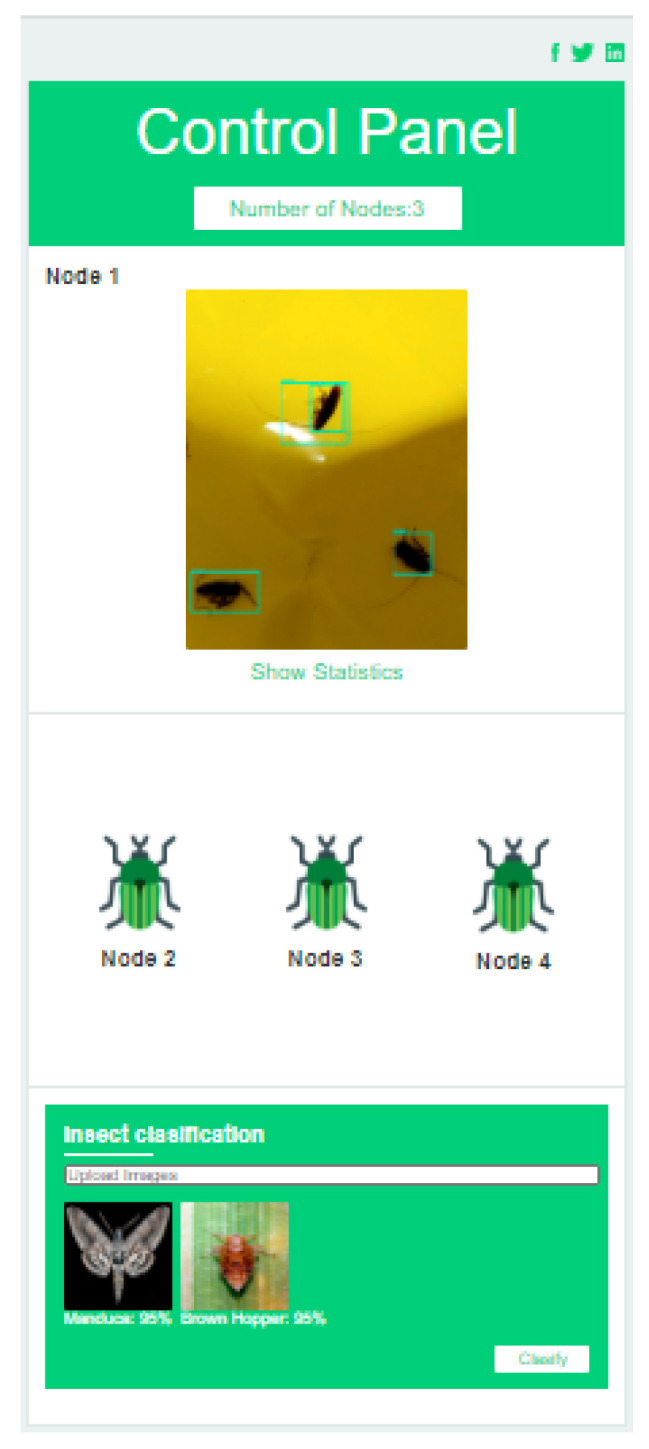
Overview of android mobile APP.

The application layer delivers the insect trap status information to the end-user. Smartphones and web interfaces are used to execute the application layer task. In this work, web-based GUI and android mobile are developed to track the status information. Figure 3 shows the layout of the android mobile application developed for insect monitoring.

## 3. Experimental Results

**Figure 4:**
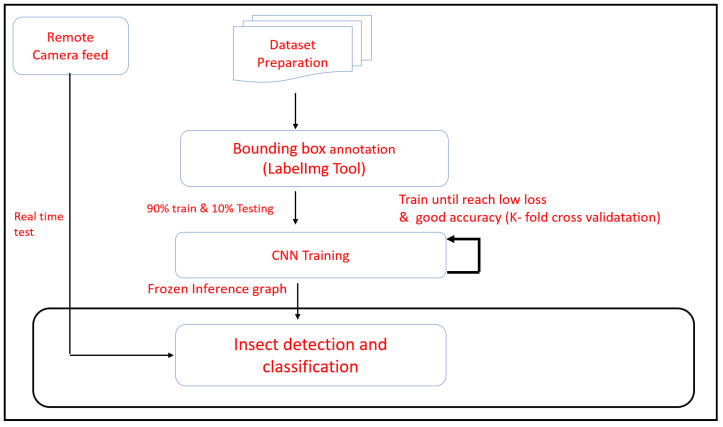
Experimental design flow.

This section describes the experimental design procedure and results of the remote insect trap monitoring and insect detection method. The Figure 4 shows the experimental design flow of the proposed system.

### 3.1. Dataset Preparation and Annotations

The dataset preparation process involves collecting the insect’s image from a different online source. Built environment lizard, crawling and flying insects ants, cockroach and flys and farm field insect Planthoppers, Colorado, Empoasca, Mole-cricket, Manduca, Rice Hispa, Stink-bug and Whiteflies were adopted for dataset preparation. In each insect class, 1000 images were used to train the model. The insect images were collected from following online insect image database include [41], IP102 [42], rice knowledge bank [43], bugwood [44]. Then, the data augmentation is applied to control the over-fitting and improve the CNN learning rate. The data augmentation process such as image rotation, flipping, and scaling was applied to collected images. The image resolution of 640×480 was used in both training and testing the CNN model. After the data augmentation process, the dataset labeling was performed using a bounding box and class annotations tool “LabelImg”. LabelImg is a Graphical User Interface (GUI) based bounding box annotation tool used to mark the insect categories and rectangular bounding boxes of the insect images. The GUI is written in Python and uses Qt for its graphical interface. Annotations are saved as XML files in PASCAL Visual Object Classes (VOC) format and also support YOLO format.

### 3.2. Hardware Details

The unified DL based insect detection model was developed in Tensor-flow 1.9 open source machine learning platform run on Ubuntu 18.04 version. The model was trained and tested on GPU enabled workstation consists of Intel core i7-8700k, 64 GB Random Access Memory (RAM), Nvidia GeForce GTX 1080 Ti Gaming Graphics Processing Unit (GPU) Card (3584 NVIDIA CUDA Cores and 11 Gbps Memory Speed). The same hardware is used to run the insect detection and classification task.

### 3.3. Training

The pre-trained ResNet 50 model was used to feature extraction task. It was trained on the ImageNet dataset. Stochastic Gradient Descent (SGD) algorithm was used for training the Faster R-CNN module (RPN and Fast RCNN). It uses a momentum of 0.9 and an initial learning rate of 0.0002 with a batch size of 1, respectively. In the RPN training phase, each iteration 128 training samples are randomly chosen from each training image. Here, the ratio between positive (object) and negative (background) samples is 1:1 ratio. Further, the original Faster RCNN value parameter was used to fine-tune the NMS.

In the training phase, the loss for each prediction is estimated as a sum of the location loss $L_{location}$ and confidence loss $L_{confidence}$ (Equation (1)). Here, the confidence loss is the fault in the prediction of object class and confidence level. The location loss is referred to as the squared distance between the coordinates of the prediction. A parameter α is adopted to equivalence the two losses and their impact on the gross loss. Root Mean Squared gradient descent algorithm is used to optimized this loss. It calculate the weights $w_{t}$ at any time *t* using the gradient of loss *L*, $g_{t}$ and gradient of the gradient $v_{t}$ (Equations (2)–(4)). Hyper-parameter β,η are used to balance the terms used for momentum and gradient estimation, while ϵ is a small value close to zero for preventing divide by zero errors.

(1) $$L=\frac{1}{N}\left(L_{confidence}+\alpha L_{location}\right)$$

(2) $$v_{t}=\beta v_{t-1}+\left(1-\beta \right)g_{t}^{2}$$

(3) $$\Delta w=\frac{-\eta }{\sqrt{v_{t}+\epsilon }}\times g_{t}$$

(4) $$w_{t+1}=w_{t}+\Delta w$$

The efficacy of the various sets of training data is determined by K-fold (here K = 10) cross-validation process. The dataset is divided into K subsets and K − 1 subsets used for training, and the remaining one subset is used for evaluating the performance. This process is run in K times to obtain the mean accuracy and other quality metrics of the detection model. K-fold cross-validation is done to verify that the images reported are accurate and not biased towards a specific dataset split. The images shown are attained from the model with good precision.

### 3.4. Evaluation Metrics

Standard statistical measures such as accuracy (Equation (5)), precision (Equation (6)), recall (Equation (7)) and $F_{measure}$ (Equation (8)) were used to assess the detection and classifier performance. The bounding box’s performance metrics are calculated by comparing the predicted bounding box with the actual bounding box. An Intersection Over Union (IOU) operation is used to determine how close the predicted bounding box is compared to the actual bounding box. The bounding box’s accuracy is directly proportional to the IOU score calculated between the actual and predicted bounding box. Therefore, a confusion matrix is generated using the IOU score obtained from the bounding box. If the IOU is above a specific threshold, then that predicted bounding box is deemed to match the true bounding box and is considered a true positive or true negative. If the IOU score is lesser than the threshold, then the predicted bounding box is deemed to be incorrect and considered a false positive or false negative. In addition to this, the overall mean IOU value for all the predicted bounding box was also calculated to determine the model’s performance. From IOU output, we can calculate the Average Precision (AP) and Recall metrics. It is a common performance indicator for evaluating the performance object detector module. Further, the performance metrics for object classification is calculated by constructing a confusion matrix using the actual and predicted image labels.

(5) $$Accuracy\left(Acc\right)=\frac{tp+tn}{tp+fp+tn+fn}$$

(6) $$Precision\left(Prec\right)=\frac{tp}{tp+fp}$$

(7) $$Recall\left(Rec\right)=\frac{tp}{tp+fn}$$

(8) $$F_{measure}\left(F_{1}\right)=\frac{2\times precision\times recall}{precision+recall}$$

Here, tp,fp,tn,fn represents the true positives, false positives, true negatives, and false negatives, respectively, as per the standard confusion matrix.

### 3.5. Offline and Real Time Test

**Figure 5:**
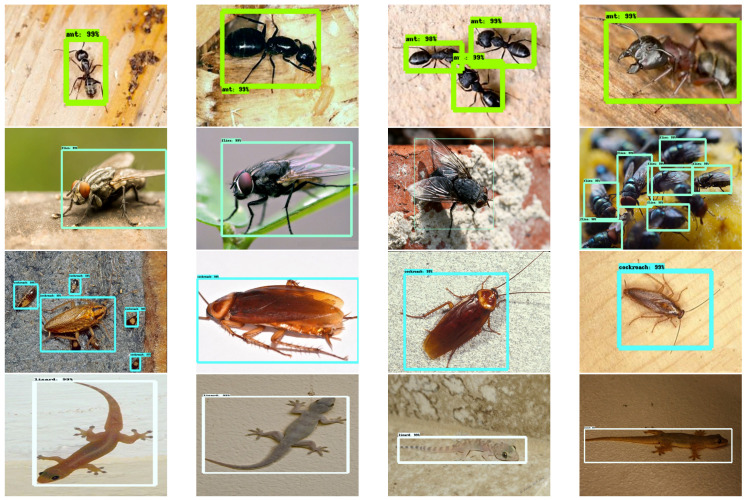
Offline test.

**Figure 6:**
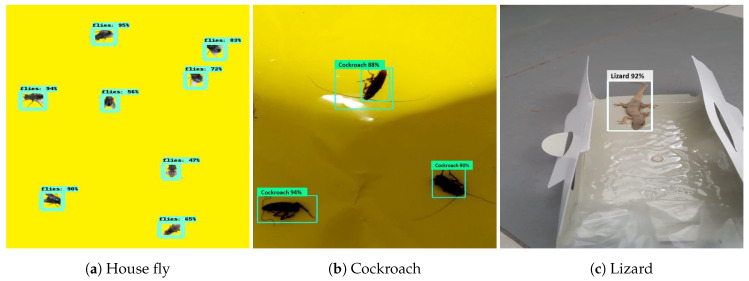
Real time test results.

There were 150 test images used for each class. For the offline test, the images are collected from image databases that images are not used for training. The detection results of the built environment lizard and insect detection are shown in Figure 5. For a real-time remote insect traps monitoring trial, the insect trap sheet was fixed in three different locations: Kitchen, food storage region, and drainage water outlet. The trap was a monitor with a Trek AI ball WiFi-enabled camera. The detailed specification of the AI ball camera is given in Table 1. The camera is fitted as downward on the wall (vertically 90 degree) to focus on the insect trap and connected with the internet through a 2.4 GHz home WiFi network for transferring the trap sheet image to the processing layer. Further, to carry out the insect detection task, the trained model was configured in GPU enabled workstation, which runs the processing layer task. The experiment was performed for 14 days continuously to track the insects detected on the trap sheets. In our trap sheet cockroach, lizard and flies were trapped, and its results are shown in Figure 6.

The detection results ensure that the insect detection model run on the IoT processing layer has accurately detected and classified the insects with a higher confidence level in offline test and real-time. Further, statistical measures have been performed for estimating the robustness of the detection model. Table 2 shows the statistical measures result for built environment lizard and insect detection for both offline and online experiments.

The table result indicates that the trained object detection framework has detected the cockroach with an average of 96.83%, a lizard with 97.68%, and house fly’s with 95.16% respectively. It is observed that the confidence level and statistical measure values for the house fly detection are slightly low compared to other classes. This outcome is understandable, as house flies have less visibility and complex texture. Further, the model’s computation time was assessed through model inference time; in this analysis, the trained model took 0.02 s for process the one 640 images.

### 3.6. Farm Field Insect Detection

**Figure 7:**
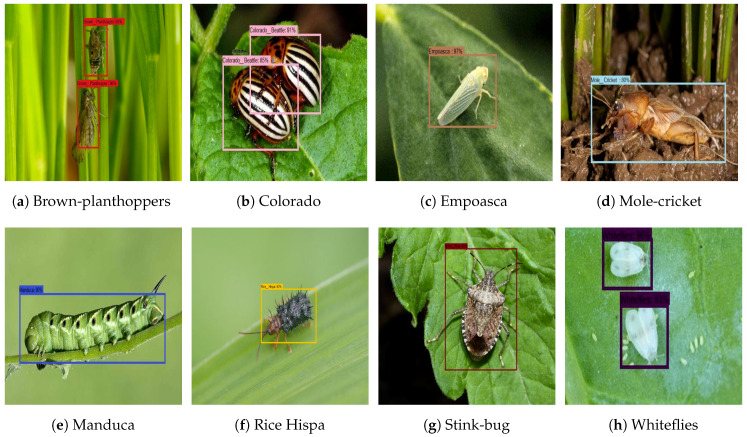
Garden and farm field Insect Identification.

For the farm field insect detection test, 150 test images are used for each class. The images are collected from the farm field insect image database. Figure 7 shows the farm field insect detection results. The experiment results show that the algorithm accurately localizes the insect with a higher confidence level. Further, accuracy, precision, recall, and F1 measure were computed to evaluate the model’s statistical performance. The statistical performance of each class is shown in Table 3. In this analysis, we observe that the trained CNN model detected and classified the farm field insect with an average of 94% accuracy.

### 3.7. Comparison with Other Object Detection Framework

**Figure 8:**
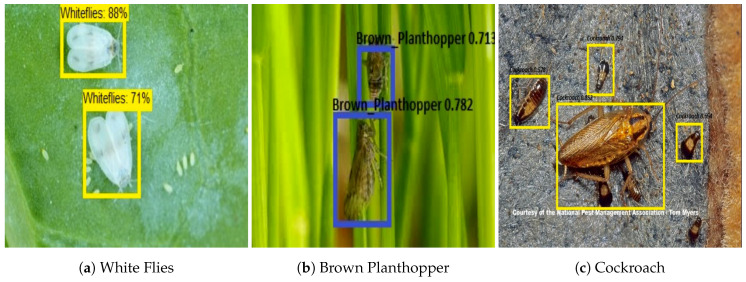
Yolo V2 insect identification results.

**Figure 9:**
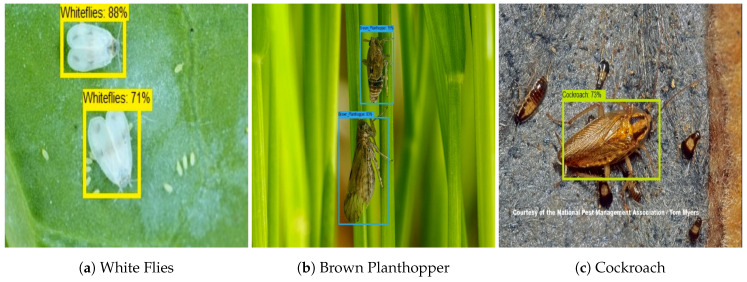
SSD MobileNet insect identification results.

**Figure 10:**
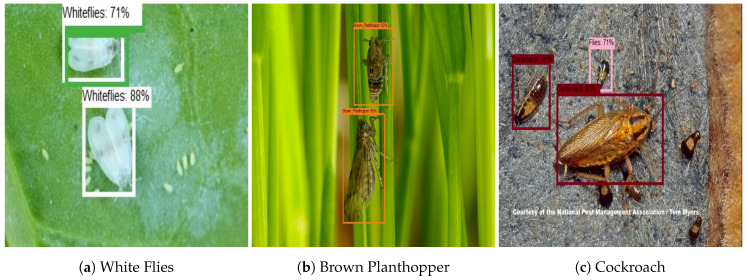
SSD inception insect identification results.

This section evaluates the performance of the proposed system with popular single-shot object detection algorithms SSD and Yolo V2. Here, MobileNet and Inception v2 [45] classifiers were used with SSD for feature extraction task [46,47,48]. Similarly, darknet19 feature extractor is used in Yolo v2 module [49,50,51]. The three detection frameworks are trained with the same lizard and insect image dataset and similar amount of training time. Figure shows the detection results of Yolo v2 (Figure 8), SSD MobileNet (Figure 9) and SSD inception (Figure 10) model.

The comparison (Table 4) results indicate that the proposed system has good accuracy in localizing the insects compare to SSD and Yolo V2 detection models. Furthermore, the classification result indicates that Yolo has a false classification, and its accuracy level is relatively low compared with other models. Similarly, miss detection and false classification ratio have slightly high in SSD MobileNet and SSD Inception trial.

Furthermore, the computational cost of both training and testing was estimated for each model. The computational cost for training was estimated using each model’s training time to reach training error is minimum, and the computational cost for the testing time was estimated by execution time per image. Table 5 shows the computational cost of both training and testing. In that analysis, we observe that minimum execution time was observed in YOLO v2 compared with the Faster RCNN and SSD. However, Faster RCNN ResNet50 obtained the best detection accuracy. In our experiment, accurate insect detection is a crucial objective. Hence, the Faster RCNN ResNet50 framework is more suitable for the insect identification field.

### 3.8. Comparison with Existing Work

This section performs the comparison analysis with an existing insect identification scheme reported in the literature. Table 6 shows the comparison report of various insect detection scheme. The comparison has been reported based on the algorithm used to identify the insect and detection accuracy.

The Table 6 results indicate that the proposed scheme obtained improved insect identification accuracy than existing farm field insect identification and built environment insect identification methods. However, comparing the table result directly with our proposed system is not fair. Because the authors used different CNN frameworks, different dataset, and various training parameters; hence, the performance cannot be accurately compared.

To the best of our knowledge, we didn’t find any IoT combined CNN based insect identification schemes in the literature. Our proposed system combines the IoT and CNN based insect identification scheme. Through, our scheme insect management systems easily monitor and identify the insects remotely without human assistance.

### 3.9. Application and Future Work

The proposed system is more useful to pest control industries for monitoring the pest in a various environment like food storage region, hospitals, and garden, etc. Further, early insect detection in farm field will reduce yield loss by up to 20–40% and also will help for to reduce the excessive use of various harmful pesticides in agriculture. The current study focuses only on built environment lizard, crawling and flying insects, and selected farm field insects. In our future work, we plan to develop the DL enabled drone and inspection class robot to detect the various types of rodents, develop phases of the built environment and farm field insects, and crop diseases.

## 4. Conclusions

Remote insect monitoring and automatic insect identification method were proposed in this paper using the IoT and deep-learning framework. Four-layer IoT framework was used to construct the remote trap insect monitoring system, and the Faster RCNN ResNet object detection framework was used to identify the insect class automatically. The efficiency of deep learning-based insect identification was tested offline and online with various insect image databases includes a built environment insect database and farm field insect database. In contrast with other object detection frameworks, including SSD and Yolo, the proposed scheme scored better insect detection accuracy. The experimental results showed that the trained model obtained 96% insect identification accuracy to built environment insects, 94% identification accuracy to farm field insect, and took an average of 0.2 s to process the one image. This case study proved that IoT and the DL based insect monitoring scheme automate the remote insect identification method through a trained CNN framework and overcomes the shortcoming of the insect management system.

## Figures and Tables

**Table 1:** Artificial Intelligence (AI) ball camera specification.

Specification	Details
View Angle	60 degree
Output image format	VGA 640 × 480, QVGA 320 × 240, QQVGA 160 × 120
Output Video format	Motion JPEG
Frame Per second (FPS)	30
Wireless Interface	IEEE 802.11b/g 2.4 GHz ISM Band
Wireless Range	20 m
Dimension/Weight	30 mm (Diameter) × 35 mm (L)/100 g
Power Supply/Consumption	Voltage: 3.0 V , Power: 750 mAH

**Table 2:** Statistical measures for insects detection.

Test	Cockroach				Lizard				Housefly			
	Prec.	Recall	$F_{1}$	Accuracy	Prec.	Recall	$F_{1}$	Accuracy	Prec.	Recall	$F_{1}$	Accuracy
offline (database)	97.21	97.12	97.04	97.00	98.64	98.18	98.25	98.31	95.77	95.44	95.29	95.33
Real time (trap sheet)	96.45	96.22	96.17	96.29	96.72	96.17	96.03	96.43	94.89	94.38	94.04	94.27

**Table 3:** Statistical measures for farm field insects identification.

Insect Name	Precision	Recall	F1	Accuracy
Planthoppers	93.25	92.39	92.11	93.13
Colorado	95.23	94.89	94.22	94.88
Empoasca	94.34	93.90	93.54	94.08
Mole-cricket	94.65	94.24	94.19	94.33
Manduca	93.05	92.93	92.74	92.97
Rice Hispa	94.21	93.82	93.77	93.88
Stink-bug	95.42	95.13	95.17	95.00
Whiteflies	94.80	94.23	94.01	94.60

**Table 4:** Comparison with other frame work using bounding box detection and classification metrics.

Test	Bounding Box Detection (IOU > 0.5)			Classification			
	Prec.	Recall	mAP	Prec.	Recall	$F_{1}$	Accuracy
Yolo V2	79.15	84.13	78.65	89.33	87.55	87.11	87.66
SSD MobileNet	84.34	87.78	82.31	92.31	92.07	92.00	92.12
SSD Inception	85.61	88.13	86.52	93.74	93.16	93.05	93.47
Proposed	90.10	89.78	88.79	96.22	95.98	95.79	96.08

**Table 5:** Computational cost analysis.

Algorithm	Computational Cost for Training (Hours: Minutes)	Computational Cost for Testing (Seconds)
Yolo V2	7:20	20.22
SSD MobileNet	7:50	15.88
SSD Inception	8:35	26.03
Proposed	9:30	31.66

**Table 6:** Comparison with other insect detection scheme.

Case Study	Application	Algorithm	Detection Accuracy
Xia et al. [31]	Farmfield	VGG19 + RPN	0.89
Nguyen et al. [30]	Pest detection on Traps	SSD + VGG16	0.86
Liu et al. [29]	agriculture pest identification	PestNet	0.75
Rustia et al. [12]	built environment insects	YOLO v3	0.92
Ding et al. [27]	farm field moth	ConvNet	0.93
Proposed system	built environment and farm field	Faster RCNN ResNet 50	0.94
